# *Arabidopsis thaliana* mutants lacking cpFtsY or cpSRP54 exhibit different defects in photosystem II repair

**DOI:** 10.3389/fpls.2015.00250

**Published:** 2015-04-13

**Authors:** Björn Walter, Thomas Pieta, Danja Schünemann

**Affiliations:** ^1^Molecular Biology of Plant Organelles, Ruhr-University BochumBochum, Germany; ^2^Plant Cell Physiology and Molecular Biology, Ruhr-University BochumBochum, Germany

**Keywords:** photosystem II, signal recognition particle, thylakoid membrane, protein transport, D1, photodamage

## Abstract

Photosystem II (PS II) is a multi subunit protein complex embedded in the thylakoid membrane of cyanobacteria and chloroplasts. As the PS II reaction center protein D1 is prone to a light induced damage that inhibits PS II function especially at elevated light intensities, a highly ordered repair process including synthesis, targeting and insertion of D1 has evolved. To elucidate the function of the chloroplast signal recognition particle subunits, cpSRP43 and cpSRP54, and the cpSRP-receptor cpFtsY in D1 biogenesis we investigated the efficiency of the PS II repair cycle in the corresponding mutants of *Arabidopsis thaliana*. Immunological analyses, PAM measurements and *in vivo* labeling experiments demonstrate an impaired replacement of damaged D1 in the *cpftsy* mutant, while the *chaos* and the *ffc* mutant lacking cpSRP43 and cpSRP54, respectively, were not or hardly affected. The defect in *cpftsy* was neither caused by an impaired *psbA* transcript accumulation, D1 translation initiation nor by an enhanced D1 degradation. Further experiments revealed a decreased amount of salt stable, thylakoid membrane-associated translating ribosomes in the *cpftsy* mutant, while the amount of membrane-associated translating ribosomes is unaltered in the *chaos* and the *ffc* mutants. Therefore, our data indicate that the lack of cpFtsY leads to an inefficient PS II repair cycle caused by an impaired binding of translating ribosomes to the thylakoid membrane.

## Introduction

The photosystem II (PS II) is a multi subunit protein pigment complex embedded in the thylakoid membrane of cyanobacteria and chloroplasts of algae and higher plants. There, it facilitates the light dependent charge separation and water splitting. The PS II subunits are encoded on the plastid and the nuclear genome. Nuclear encoded PS II subunits are synthesized in the cytosol and imported into the chloroplast posttranslationally (Schleiff and Becker, 2011; Paila et al., 2014). After the import into the stroma, the PS II subunits are transported across or inserted into the thylakoid membrane by four distinct mechanisms (Schünemann, 2007; Albiniak et al., 2012; Celedon and Cline, 2013). One of these is the posttranslational chloroplast signal recognition particle (cpSRP) dependent pathway that is responsible for the insertion of the light harvesting chlorophyll a/b binding proteins (LHCPs) that form the major antenna complexes of both photosystems. The cpSRP comprising the 43 kDa subunit cpSRP43 and the universally conserved 54 kDa subunit cpSRP54 binds the highly hydrophobic LHCPs to keep them soluble during the passage through the stroma. This transit complex is recruited to the thylakoid membrane by the membrane-associated receptor cpFtsY and the insertase Alb3, which is responsible for LHCP insertion (Richter et al., 2010). In contrast to nuclear encoded PS II subunits, plastid encoded polytopic PS II subunits are cotranslationally inserted into the thylakoid membrane. In *Escherichia coli*, translating ribosomes (RNCs) synthesizing polytopic inner membrane proteins are guided to the plasma membrane in a concerted process facilitated by the SRP consisting of the cpSRP54 homolog Ffh and the SRP RNA (Akopian et al., 2013). Ffh binds RNCs by a direct interaction with the ribosomes exit tunnel and the nascent polypeptide chain (Gu et al., 2003; Bornemann et al., 2008). Subsequently, this SRP-RNC complex is guided to the SecYEG translocase by an interaction with the membrane associated receptor FtsY (Akopian et al., 2013; Denks et al., 2014). In chloroplasts, cpSRP54 interacts transiently with the nascent D1 chain (Nilsson et al., 1999; Nilsson and van Wijk, 2002) and D1 was shown to be inserted into the thylakoid membrane via the cpSec-translocon (Zhang et al., 2001). However, the chloroplast SRP system in higher plants differs significantly from the bacterial SRP-dependent transport as it does not contain a SRP-RNA, which is indispensable in all cytosolic SRP systems (Schünemann et al., 1998; Träger et al., 2012).

As the D1 protein is prone to a light induced damage, the replacement of D1 is a critical step during the PS II repair cycle to maintain its functionality (Kato and Sakamoto, 2009; Nixon et al., 2010). Previously, analyses of *Arabidopsis thaliana* mutants lacking cpSRP43 (*chaos*), cpSRP54 (*ffc*) and cpFtsY (*cpftsy*) demonstrated that *chaos* was characterized by a specific defect in accumulation of LHC proteins while *ffc* and *cpftsy* showed in addition a reduced steady state level of the reaction center proteins D1, PsaA and PsaB (Amin et al., 1999; Klimyuk et al., 1999; Tzvetkova-Chevolleau et al., 2007). However, *cpftsy* exhibited a significantly stronger phenotype than *ffc* resulting in a strong chlorotic phenotype (Tzvetkova-Chevolleau et al., 2007) and seedling lethality (Asakura et al., 2004, 2008). *Cpftsy* showed also a more severe reduction of the maximal photochemical efficiency of PS II, which indicates a defect in proper assembly and activity of PS II, and a more rapid saturation of the photosynthetic electron transport by light (Tzvetkova-Chevolleau et al., 2007). Hence, biochemical data and mutant analysis indicate a role of cpSRP54 and cpFtsY in cotranslational insertion of D1 and other chloroplast encoded membrane proteins, but their individual relevance for this process and their precise molecular function is not clear yet.

In the present study, we compared the efficiency of D1 replacement during the PS II repair cycle in the cpSRP pathway mutants and wild type. Our data reveal a drastic defect of the *cpftsy* mutant in the PS II repair cycle, while the *ffc* mutant is only slightly affected. Additionally, the *cpftsy* mutant showed a reduced association of translating ribosomes with the thylakoid membrane indicating a disturbance in the binding of D1 translating ribosomes to the membrane.

## Material and methods

### Plant materials and growth conditions

The *A. thaliana* knock out mutants lacking cpSRP54 (*ffc* 1-2, referred to as *ffc*), cpSRP43 (*chaos*), and cpFtsY (*cpftsy*) were described previously (Amin et al., 1999; Klimyuk et al., 1999; Tzvetkova-Chevolleau et al., 2007). Mutant and wild type (ecotype Col-0) plants were grown on soil under artificial light (8 h, 120 μmol photons m^−2^ s^−1^) and constant temperature of 22°C/19.5°C (day/night) in GroBanks (CLF Plant Climatics).

### Protein extraction, SDS-/BN-PAGE and immunoblot analysis

Total protein extracts of rosette leaves were prepared using the peqGOLD TriFast reagent (Peqlab). The protein concentration of the extracts was determined using the BCA protein assay kit (Pierce). Proteins were separated on 12–15% SDS-polyacrylamide gels and subsequently coomassie stained or blotted onto nitrocellulose membrane (Macherey&Nagel). Transferred proteins were detected by specific antibodies against D1 (Agrisera), Cytochrome f (Agrisera) and Rpl4 (Uniplastomic) and enhanced chemiluminescence reaction, ECL (Pierce).

BN-PAGE was conducted according to Schagger and von Jagow (1991) using the NativePAGE running buffer kit (Life Technologies). After BN-PAGE, the separated protein complexes were transferred to PVDF membrane (Macherey and Nagel) by western blot. Subsequently, the dye was removed from the membrane by incubation in methanol and the membrane was then used for immunodetection.

### Isolation of thylakoid membranes

Leaves of *A. thaliana* in the rosette stage were homogenized in 50 mM HEPES pH 8.0, 330 mM sorbitol, 15 mM Mg-acetate (SHM) supplemented with 5 mM ascorbate and 0.05% (w/v) BSA using an Ultrathurrax (IKA). The homogenate was filtered through two layers of miracloth (Calbiochem). Afterwards, the chloroplasts were harvested by centrifugation (1500 g, 5 min, 4°C) and washed in SHM. Then, chloroplasts were lysed in 20 mM HEPES pH 8.0, 15 mM Mg-acetate (HM) for 10 min on ice. After that, thylakoids were spun down (1500 g, 5 min, 4°C) and the supernatant was discarded. For BN-PAGE analyses, the pellet was resuspended in 25 mM BisTris-HCl pH 7.0, 30% (v/v) glycerol supplemented with 1.5% (w/v) β-D-dodecylmaltoside at a chlorophyll concentration of 1 mg ml^−1^ and incubated for 10 min on ice. After removal of insoluble material (20,000 g, 10 min, 4°C), the supernatant was supplemented with coomassie G-250 (Life Technologies) to a final concentration of 0.25% (w/v) and used for BN-PAGE with subsequent western blot.

To analyze the membrane association of ribosomes, the isolated thylakoids were resuspended in HM at a chlorophyll concentration of 1 mg ml^−1^ and divided into five equal fractions. The thylakoids were spun down (1500 g, 5 min, 4°C) and resuspended in HM supplemented with 60 mM NaCl, 1 M NaCl, 50 mM puromycin/60 mM NaCl, 50 mM puromycin/1 M NaCl or 0.2 M Na_2_CO_3_, respectively. After an incubation (30 min, 37°C, rotating end over end), the thylakoids were sedimented and liberated from the supernatant. Finally, the pellets were resuspended in HM at a chlorophyll concentration of 2 mg ml^−1^ and 1:1 diluted by the subsequent addition of SDS sample buffer.

### Nucleic acid analysis

The isolation of total RNA from *A. thaliana* leaf tissue was done according to US patent 5,973,137, Gentra Systems, Purescript® (Gentra-Systems Inc., Minneapolis, USA) and the concentration of RNA was determined with Nanodrop 2000c (Peqlab). For northern blot analyses, 3 μg total RNA were loaded on each lane and separated on a denaturating 1.2% (w/v) agarose gel. Subsequently, RNA was transferred onto a positively charged nylon membrane (Roth) and *psbA* transcript was detected with a specific probe.

For production of the *psbA* probe, the template vector pGEM-T Easy *psbA* (Loschelder et al., 2006) was linearized with PstI and used for *in vitro* transcription (Promega T7 transcription kit) and labeled with digoxygenin (DIG) according to the Kit manual (Roche). Hybridized probes were detected using a DIG-specific, alkaline phosphatase conjugated antibody (Roche). Chemiluminescence was detected by exposure to a film (Fuji).

Polysomes were isolated from *A. thaliana* leaves according to Barkan (1993). Leaf tissue (400 mg) was frozen in liquid nitrogen and homogenized with a mortar and a pestle. Membranes were subsequently solubilized with 1% (v/v) Triton X-100 and 2% (v/v) polyoxyethylene (10) tridecyl ether. Microsomal membranes were then solubilized with 0.5% (w/v) sodiumdeoxycholate and insoluble material was removed (15,000 g, 15 min, 4°C). The supernatant was then loaded onto a 15–55% sucrose step gradient and centrifuged [250,000 g, 65 min, 4°C, TST 60.4 rotor (Kontron)]. The gradient was fractionated into 14 equal fractions and each fraction was subjected to RNA extraction with phenol-chloroform two times. After ethanol precipitation, the RNA was subjected to northern blot analysis as described above. To mimic the situation of impaired translation initiation of *psbA* mRNA, the polysomes were disrupted according to Lu and Hanson (1996).

### Chlorophyll fluorescence analysis

The maximum quantum efficiency F_v_ F^−1^_m_ of intact leaves of *A. thaliana* null mutants and Col-0 was determined using a portable PAM-2000 (Heinz Walz GmbH, Germany, www.walz.com). Before measurements, each plant was dark adapted for 15 min.

### *In vivo* labeling of proteins and pulse-chase experiments

The *de novo* synthesis of thylakoid membrane proteins was monitored as described previously (Lennartz et al., 2001) with the following modifications. Leaf discs of *A. thaliana* plants in rosette stage where prepared using a cork drill (0.5 cm diameter). The leaf discs were incubated for 30 min at RT in preparation buffer (50 mM Tris-HCl pH 7.5, 0.2 mg ml^−1^ cycloheximide and 0.1% (v/v) Tween-20). Subsequently, the leaf discs were transferred to labeling buffer (50 mM Tris-HCl pH 7.5, 0.1 mg ml^−1^ cycloheximide, 0.1% (v/v) Tween-20 and 0.1 μCi μ^−1^ [^35^S]-methionine (specific activity >1000 Ci mmol^−1^, Hartmann Analytic) and incubated at 25°C for 60 min at indicated light intensities. After washing the leaf discs five times with washing buffer (50 mM Tris-HCl pH 7.5, 750 mM NaCl, 2 mM EDTA), the tissue was homogenized with a steel pestle in 300 μl homogenization buffer (50 mM Tris-HCl pH 7.5, 150 mM NaCl, 2 mM EDTA). Next, membranes were pelleted (15,000 g, 10 min, 4°C) and washed two times. Subsequently, membranes were resuspended in 50 μl homogenization buffer supplemented with 2% (w/v) SDS and incubated for 10 min at 25°C with a following heating at 70°C for 2 min. After centrifugation (10 min, 15,000 g, RT), the supernatant was transferred to a new reaction tube. The supernatant was used for chlorophyll determination and SDS-PAGE and radiolabeled proteins were detected by autoradiography.

For pulse-chase experiments, chloroplast proteins in leaf discs were pulse labeled as described above for 60 min followed by a chase time in 10 mM non-labeled L-methionine for 8 h at 150 μmol photons m^−2^ s^−1^. Every hour, five leaf discs were frozen in liquid nitrogen and homogenized in homogenization buffer supplemented with 1 M NaCl. After centrifugation, the membrane proteins were SDS-solubilized in 40 μl homogenization buffer as described above. Subsequently, 15 μl were used for SDS-PAGE.

### Quantification

Signals of western and northern blots and radiolabeled proteins were quantified using ImageJ (http://imagej.nih.gov/ij).

## Results

### The *cpftsy* mutant is drastically impaired in PS II repair while the *ffc* mutant is only slightly affected

**Figure 1:**
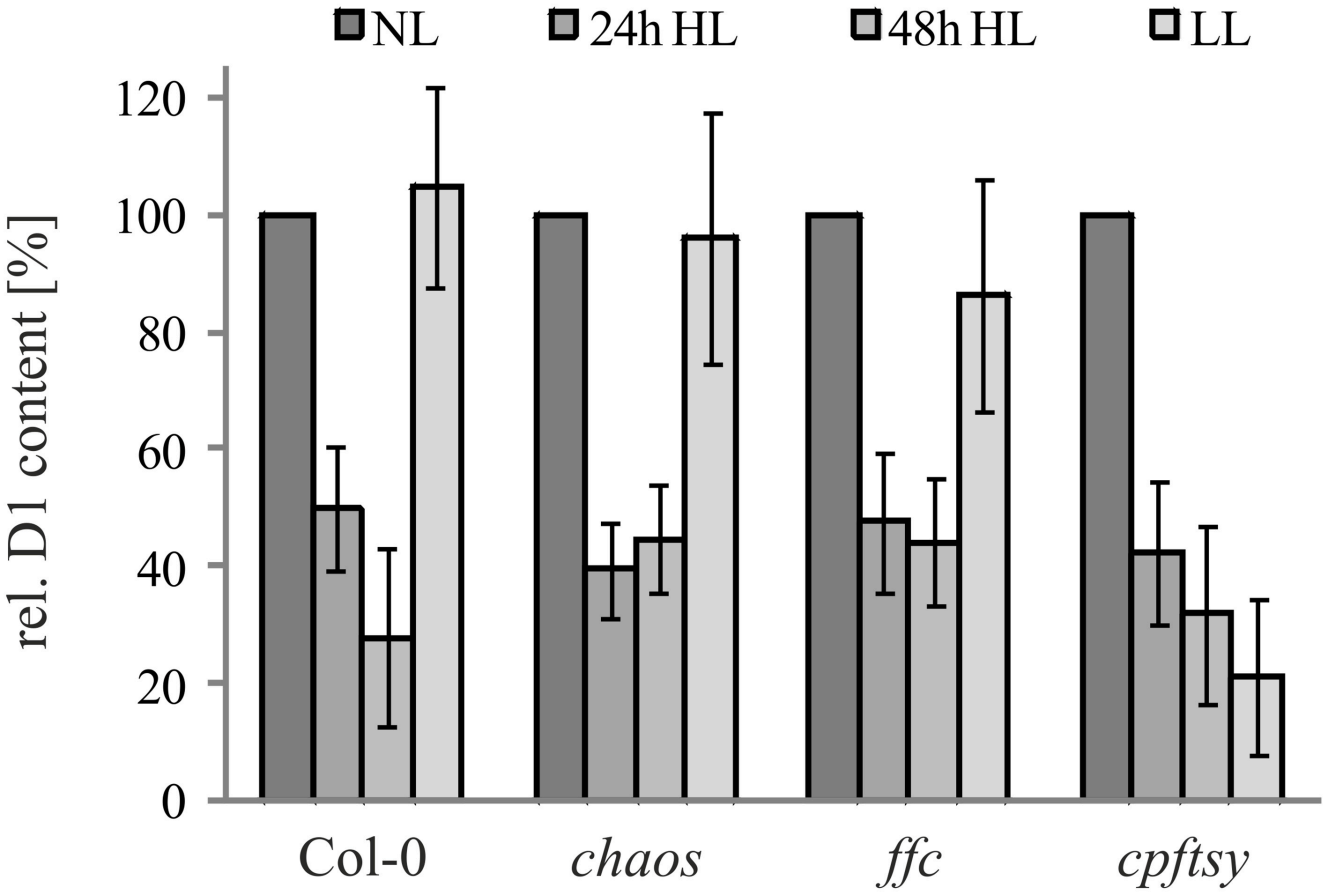
**D1 level in cpSRP mutant plants under high light conditions and a subsequent recovery phase**. Wild type and cpSRP mutant plants were raised at normal light conditions (NL, 120 μmol photons m^−2^ s^−1^) to rosette stage and then exposed to high light (HL, 1000 μmol photons m^−2^ s^−1^) for 24 or 48 h. Subsequently, plants were exposed to low light for 48 h (LL, 50 μmol photons · s^−1^ · m^−2^). Total protein extracts from 100 mg fresh weight of intact and fully expanded leaves were prepared after each light treatment and used for immunodetection of D1. The signals of the D1 immunoblots were quantified using the ImageJ program. The D1 levels in normal light conditions were set to 100%. Error bars indicate the standard deviation (*n* = 4).

To examine the role of cpSRP43, cpSRP54, and cpFtsY in the biogenesis of plastid-encoded D1, we initially compared the D1 protein level in the *chaos*, *ffc*, and *cpftsy* mutants and wild type plants after growth at normal light conditions (120 μmol photons m^−2^ s^−1^), high light stress (1000 μmol photons m^−2^s^−1^) and a following recovery phase at low light conditions (50 μmol photons m^−2^ s^−1^) by western blot analysis (Figure 1). The D1 protein level decreased drastically after growth at high light conditions to about 30–40% of the initial protein level in all analyzed plant lines. After the recovery phase the amount of D1 in the wild type and the *chaos* mutant recovered completely. Interestingly, the D1 protein level in the *ffc* mutant recovered up to 90% of the initial level, while the D1 protein level in *cpftsy* showed no recovery.

**Figure 2:**
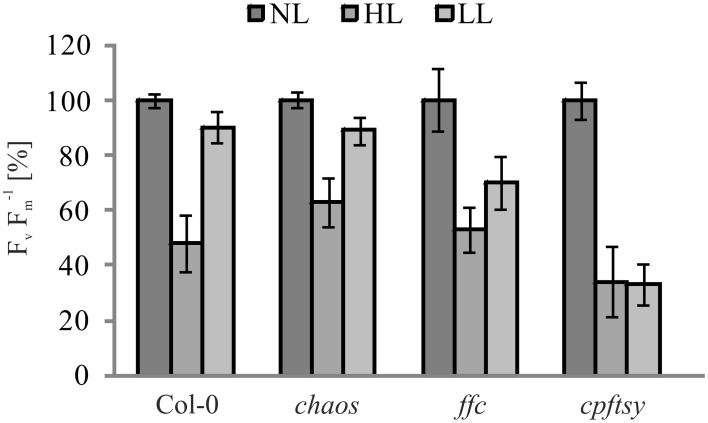
**Maximum quantum efficiency of PS II in cpSRP mutants**. The maximum quantum efficiency of PS II (F_v_ F^−1^_m_) was determined in wild type and mutant plants in the rosette stage after dark adaption. Prior to the measurements, plants were raised under normal light conditions (NL, 120 μmol photons m^−2^ s^−1^), then exposed to high light for 180 min (HL, 1000 μmol photons m^−2^ s^−1^) with a following regeneration at low light for 90 min (LL, 50 μmol photons m^−2^ s^−1^). For each plant, F_v_ F^−1^_m_values at NL were adjusted to 100%. The error bars show the standard deviation (*n* = 10).

As the *de novo* insertion of D1 is a major step in the maintenance of PS II, we used pulse amplitude modulation (PAM) measurements to analyze chlorophyll a fluorescence of P680 in order to examine the maximum quantum efficiency (F_v_ F^−1^_m_), an indicator for PS II functionality. To this end, wild type and mutant plants were sequentially exposed to normal light, high light and low light. Prior to each measurement, plants were dark adapted for 15 min. The PAM measurements revealed a decline of maximum quantum efficiency after exposure to high light in all analyzed plants. After the subsequent low light period, the maximum quantum efficiency in wild type and *chaos* recovered almost completely to about 90% of the initial value. In agreement with our previous observation, the recovery efficiency in *ffc* and *cpftsy* differed significantly. While the *ffc* mutant showed a recovery of up to 70% of its initial value, the *cpftsy* mutant showed no recovery of the maximum quantum efficiency (Figure 2).

**Figure 3:**
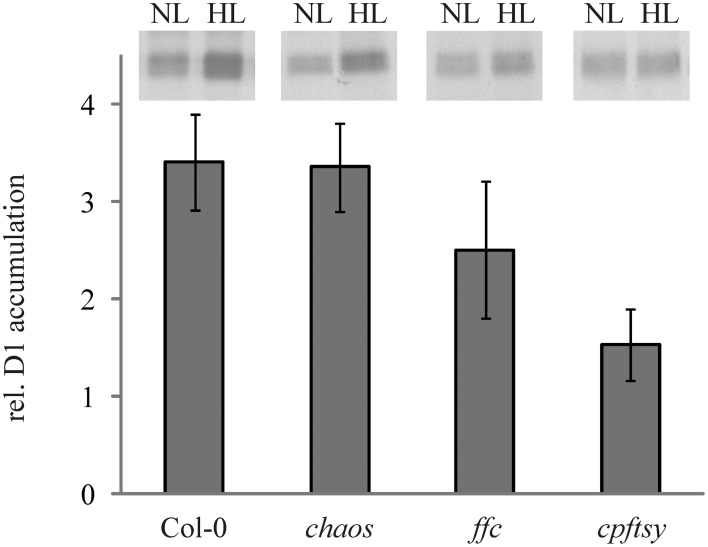
***In vivo* synthesis ratio of [^35^S]-labeled D1 in wild type and cpSRP mutant plants at high light compared to low light**. Leaf discs of wild type and cpSRP mutant plants were incubated in [^35^S] methionine containing solution at low light (50 μmol photons m^−2^ s^−1^) and higher light (400 μmol photons m^−2^ s^−1^) after preincubation in cycloheximide for 60 min. Subsequently, thylakoid membrane proteins were extracted and used for SDS-PAGE and autoradiography (equal amounts of chlorophyll were loaded). At the top, representative autoradiographs of radiolabeled D1 are depicted. The signals were subsequently quantified using the ImageJ program and the higher light values were related to low light values. A value of 1 indicates that no elevated D1 accumulation was observed under higher light conditions in comparison to low light. The error bars show the standard deviation (*n* = 4).

Growth at high light conditions is accompanied by a higher synthesis rate of D1 to enable an effective PS II repair cycle. To compare the ability of the cpSRP mutants and the wild type to increase the D1 synthesis rate at high light intensities, leaf discs were incubated in a medium containing radiolabeled methionine at low and higher light intensities of 50 and 400 μmol photons m^−2^ s^−1^, respectively. As shown in Figure 3, wild type and *chaos* accumulated about 3.5 times more radiolabeled D1 at elevated light intensities in comparison to low light, while the D1 synthesis in *ffc* was elevated 2.5 fold compared to low light. In contrast, *cpftsy* showed only a very slight increase by a factor of 1.5 in D1 accumulation.

**Figure 4:**
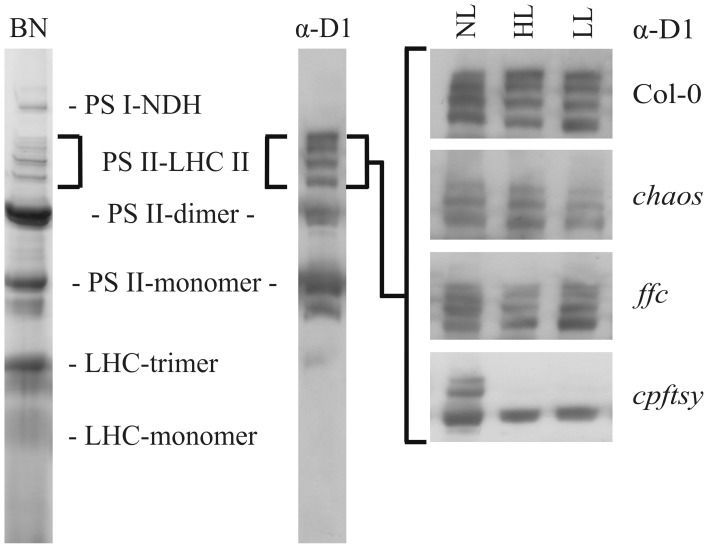
**Abundance of PS II-LHC II supercomplexes in wild type and cpSRP mutants at various light conditions**. Thylakoid membrane protein complexes of wild type and cpSRP mutants were solubilized with 1.5 (w/v) β-D-dodecylmaltoside after growth at normal light (NL, 120 μmol photons m^−2^ s^−1^), high light (HL, 1000 μmol photons m^−2^ s^−1^ for 16 h) and a subsequent regeneration at low light (LL, 50 μmol photons m^−2^ s^−1^ for 8 h). Of each sample, an equal amount of chlorophyll (1.5 μg) was separated by BN-PAGE and blotted onto PVDF membrane. The amount of PS II-LHC II super complexes was analyzed using a D1 specific antibody.

To further analyze the role of the cpSRP components in D1 biogenesis, we investigated the ability of the *chaos*, *ffc*, and *cpftsy* mutants and the wild type to maintain high molecular weight PS II-LHC II supercomplexes at elevated light conditions. To this end, we conducted BN-PAGE analyses of thylakoid membrane protein complexes of plants exposed to normal light conditions, high light intensities and a subsequent recovery at low light conditions. The separated thylakoid membrane protein complexes were subsequently analyzed immunologically to detect D1 protein containing complexes. After growth at normal light conditions, clear signals of PS II-LHC II supercomplexes were detected in *chaos*, *ffc*, *cpftsy*, and wild type plants (Figure 4). However, the composition of the supercomplexes differed in *chaos* and *cpftsy* from the wild type, which is likely due to variable compositions of the antenna complexes caused by the impact of the mutations on posttranslational LHCP transport. In addition, we observed a difference between the supercomplexes of *cpftsy* compared to *chaos* indicating an additional defect in PS II-LHC II biogenesis in *cpftsy*. Notably, the most drastic difference in PS II-LHC II supercomplex composition was observed at high light conditions between *cpftsy* and the other plant lines. At high light, the PS II-LHC II supercomplexes in wild type, *chaos* and *ffc* were hardly affected. Contrary, the *cpftsy* mutant showed a drastic reduction of PS II-LHC II supercomplexes without any regeneration during subsequent exposure to low light.

Taken together, our data provide several lines of evidence indicating a severely reduced PS II repair efficiency in *cpftsy*, while *ffc*, and *chaos* are only slightly and not affected, respectively.

### *psbA* transcript accumulation, polysome formation and D1 stability are not affected in the *cpftsy* mutant

**Figure 5:**
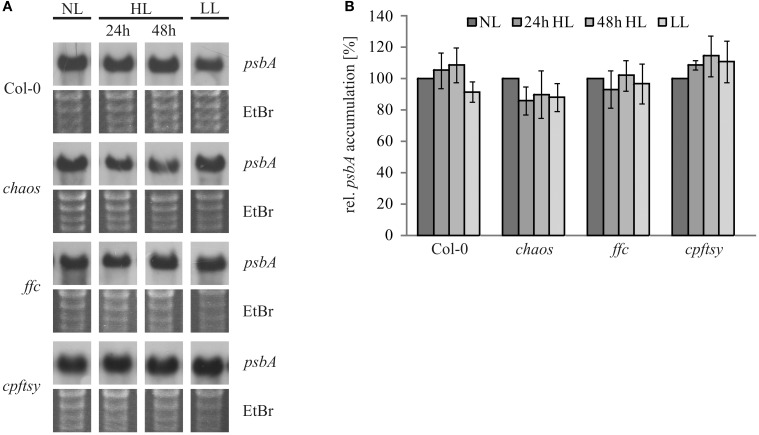
***PsbA* transcript levels during high light stress treatment and a recovery phase**. **(A)** Total RNA was isolated from fully expanded leaves of wild type and cpSRP mutant plants after growth at normal light (120 μmol photons m^−2^ s^−1^), 24 h of high light (1000 μmol photons m^−2^ s^−1^), 48 h of high light and a subsequent regeneration for 48 h at low light (50 μmol photons m^−2^ s^−1^). 3 μg of the total RNA was used for northern blot analysis to investigate the amount of *psbA* transcript. As a loading control, gels were stained with ethidiumbromide. **(B)** Northern blot signals of *psbA* transcript were quantified using the ImageJ program. The *psbA* levels at normal light conditions were adjusted to 100%. The error bars indicate the standard deviation (*n* = 3).

The reduced repair of damaged D1 in *cpftsy* suggested a possible defect in the D1 targeting or insertion mechanism. However, to rule out a high light dependent decline of the *psbA* transcript abundance, the *psbA* transcript level was analyzed in *chaos*, *ffc*, *cpftsy*, and wild type plants treated in the same way as for the investigation of the D1 protein level described in Figure 1. As shown in Figure 5, the *psbA* transcript levels of all analyzed plants were comparable under normal light and no significant changes in high light and a subsequent recovery phase were observed. Therefore, these data clearly demonstrate that the reduced PS II repair cycle in *cpftsy* is not due to a change in *psbA* transcript level.

**Figure 6:**
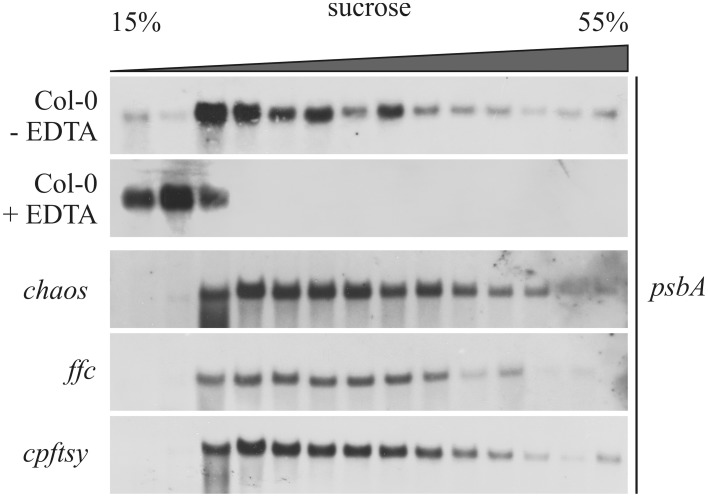
**Association of *psbA* transcript with chloroplast polysomes**. Polysomes isolated from wild type (Col-0) and mutant plants at rosette stage were fractionated on a 15–55% sucrose gradient by ultracentrifugation. Subsequently, the gradients were fractionated and the amount of *psbA* transcript was investigated in each fraction by northern blot analyses using a *psbA*-specific probe. As a control, polysomes were destroyed using EDTA.

Next, we analyzed the formation of *psbA* mRNA-associated plastid polysomes to exclude an impaired translation initiation of *psbA* mRNA. To this end, a total leaf extract of each plant line was loaded onto sucrose density gradients. Northern blot analysis of the fractionated gradient loaded with wild type extract showed that *psbA* mRNA entered fractions of higher sucrose concentration indicating an association with polysomes (Figure 6). To mimic the situation of an impaired translation reaction, a wild type leaf extract was prepared in presence of EDTA that leads to disassembly of ribosomes by depletion of Mg^2+^ (Yamamoto et al., 2010). In the corresponding sucrose density gradient, *psbA* mRNA was only detectable in fractions of low density. This reflects the situation in an *A. thaliana* mutant lacking the HCF173 protein that was shown to be essential for *psbA* translation initiation (Schult et al., 2007). The analyses of leaf extracts from the *chaos*, *ffc* and *cpftsy* mutants revealed a wild type-like distribution of *psbA* mRNA in the gradients for *chaos* and *cpftsy.* The *ffc* mutant exhibits differences in the distribution of *psbA* mRNA in fractions of higher sucrose density as less transcript was detected in these fractions (Figure 6). As it was shown previously that cpSRP54 is a component of D1-translating ribosomes, the lack of cpSRP54 might lead to a marked reduction in the molecular weight of heavy polysomes. In conclusion our data demonstrate a functional formation of polysomes and therefore an undisturbed translation initiation of *psbA* in *chaos* and *cpftsy* and indicate that this is also the case in *ffc*.

**Figure 7:**
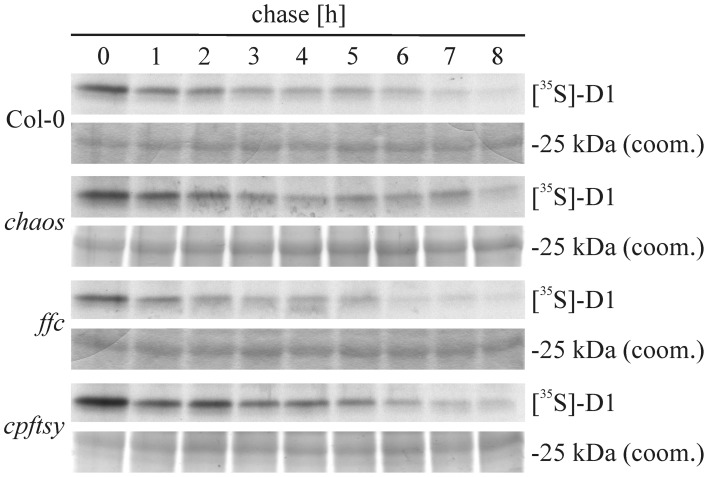
**Pulse and chase labeling of thylakoid membrane proteins**. Tylakoid membrane proteins were radiolabeled with [^35^S]-methionine in leaf discs of wild type and cpSRP mutants in presence of cycloheximide. After a pulse of 60 min, the protein turnover was chased for up to 8 h. Every hour, a sample was taken and thylakoid membrane proteins were extracted, subjected to SDS-PAGE, coomassie stained and visualized by autoradiography.

Next, we aimed to analyze whether the *cpftsy* mutant exhibits a possible enhanced degradation of D1. Therefore, pulse-chase experiments were conducted to analyze the half-time of the D1 protein in *chaos*, *ffc*, and *cpftsy* mutant. For this purpose, leaf discs of the mutant and wild type plants were incubated at low light conditions in a solution containing radiolabeled methionine and cycloheximide to label newly synthesized D1 protein and block the cytosolic translation machinery, respectively. As shown in Figure 7, clear signals of the D1 protein were still detected after a chase time of 8 h in the wild type indicating a rather high stability of the D1 protein. A similar high stability of the D1 protein was observed in the *chaos* and *cpftsy* mutants. The degradation pattern in the *ffc* mutant indicates a slightly reduced stability after 6 h of chase incubation. As our data demonstrate a high stability of the D1 protein in the *cpftsy* mutant, an enhanced D1 degradation as an explanation for the poor PS II repair cycle in *cpftsy* can be excluded.

### The cpftsy mutant is impaired in binding of translating ribosomes to the thylakoid membrane

**Figure 8:**
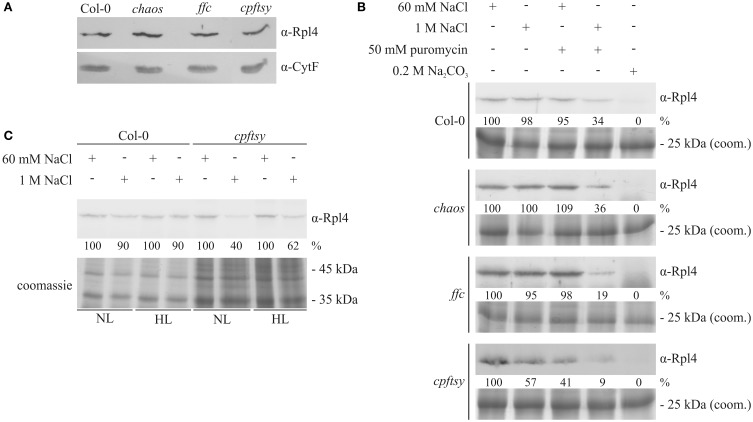
**Detection of salt-resistant and thylakoid membrane-associated ribosomes. (A)** Thylakoid membranes were isolated from wild type and the cpSRP-mutant plants grown at normal light intensity (120 μmol photons m^−2^ s^−1^). Subsequently, the thylakoids were treated with 60 mM NaCl. Afterwards, a sample corresponding to 15 μg chlorophyll was subjected to SDS-PAGE with subsequent western blot. The amount of membrane-associated ribosomes was determined using an antibody raised against Rpl4. To control the equal loading, the amount of the luminal protein Cytochrome f was investigated. **(B)** Thylakoid membranes were isolated from normal light (120 μmol photons m^−2^ s^−1^) adapted wild type and cpSRP mutants. Subsequently, the thylakoids were divided in 5 equal fractions and treated with washing conditions as indicated above. Afterwards, a sample corresponding to 15 μg chlorophyll after each washing condition was separated by 12% SDS-PAGE. Finally, the proteins were transferred onto nitrocellulose membranes and the large ribosomal subunit protein Rpl4 was detected using a specific antibody. The signals were quantified using the ImageJ program. To control the equal loading, thylakoid membrane proteins corresponding to 7.5 μg chlorophyll were separated by SDS-PAGE and visualized by coomassie staining. **(C)** Wild type and *cpftsy* plants were exposed for 8 h to normal light (120 μmol photons m^−2^ s^−1^) and high light conditions (1000 μmol photons m^−2^ s^−1^). Subsequently, thylakoid membranes were isolated and washed with lysis buffer supplemented with 60 mM NaCl or 1 M NaCl. Thylakoid membrane proteins corresponding to 15 μg chlorophyll were separated by 12% SDS-PAGE, transferred onto nitrocellulose and the ribosomal Rpl4 protein was detected using a specific antibody. The signals were quantified using the ImageJ program. To control the equal loading, thylakoid membrane proteins corresponding to 7.5 μg chlorophyll were separated by SDS-PAGE and visualized by coomassie staining.

Since bacterial FtsY is essential for the targeting of translating ribosomes to the plasma membrane (Seluanov and Bibi, 1997; Herskovits and Bibi, 2000), it was conceivable that cpFtsY might play an important role in guiding translating ribosomes to the translocon in the thylakoid membrane. Initially, we compared the amount of total ribosomes in thylakoid membranes of the cpSRP-mutants and wild type, that were purified under low-salt conditions (60 mM NaCl) by western blot analysis using an anti-Rpl4 antibody. As shown in Figure 8A, the analyzed plants exhibit a comparable amount of plastid ribosomes in the thylakoid membrane fraction. The equal loading was confirmed using an antibody raised against Cytochrome f that was shown to be imported into the thylakoid lumen independent of cpSRP (Röhl and van Wijk, 2001). To compare the amount of membrane-bound translating ribosomes in wild type and the cpSRP mutants isolated thylakoid membranes were washed with buffers containing 60 mM or 1 M NaCl with or without 50 mM puromycin. As shown in Figure 8B, the ribosomes in wild type, *chaos* and *ffc* could only be removed from the membrane by washing the membranes with a combination of high salt and puromycin. Washing with either 1 M NaCl or 50 mM puromycin did not reduce the amount of bound ribosomes. This indicates that these ribosomes are attached to the membrane by electrostatic interactions and the cotranslational insertion of nascent polypeptides into the membrane. In contrast, about 40 or 60% of the ribosomes in *cpftsy* could be detached from the membrane by washing with high salt or puromycin, respectively. This indicates that the ribosomes in *cpftsy*, which were copurifed with thylakoids under low-salt conditions, are largely inactive or non-membrane protein translating ribosomes and are therefore less tightly bound. In all analyzed plants, ribosomes as peripheral components were efficiently removed by 0.2 M Na_2_CO_3._

Next, we aimed to dissect the abundance of membrane protein translating ribosomes at the thylakoid membrane after exposure to high light conditions. Therefore, thylakoid membranes were isolated from *cpftsy* and wild type plants exposed to normal and high light and washed with 60 mM NaCl or 1 M NaCl. At both light conditions immunological analyses revealed a decrease of membrane-bound ribosomes after high salt treatment to 40–60% of the initial level exclusively in the *cpftsy* mutant, while the wild type retained 90% of the initial level (Figure 8C).

## Discussion

In this study, the question regarding the role of cpSRP43, cpSRP54 and cpFtsY during the PS II repair cycle was addressed using corresponding mutants of *A. thaliana*. Therefore, the mutant plants were exposed to various light conditions and the efficiency of PS II maintenance was monitored. A drastic limitation of the PS II repair cycle in the *cpftsy* mutant was observed that was neither due to an impaired *psbA* mRNA transcript accumulation and translation initiation nor to an enhanced D1 degradation. Instead, the amount of salt-resistant membrane-associated ribosomes was decreased in the *cpftsy* mutant. The *chaos* mutant and, notably, the *ffc* mutant showed no or only minor defects in PS II maintenance, respectively.

Based on the previous findings (1) that the *ffc* mutant shows a reduced level of the chloroplast encoded photosystem I and II reaction center proteins (Amin et al., 1999), (2) that cpSRP54 interacts transiently with the nascent chain of D1 when it emerges from the ribosome exit tunnel (Nilsson et al., 1999; Nilsson and van Wijk, 2002), (3) that one pool of cpSRP54 is associated with chloroplast ribosomes (Franklin and Hoffman, 1993; Schünemann et al., 1998) and (4) that cpSRP54 shows a strong homology to bacterial Ffh (Franklin and Hoffman, 1993), it has been assumed that cpSRP54 has a role in cotranslational protein insertion into the thylakoid membrane.

However, the lack of cpSRP54 in *A. thaliana* leads to a mild phenotype which is most visible at the young seedling stage when the first true leaves are yellowish and the subset of photosystem proteins that is reduced in the young leaves recovers to wild type level in the older leaves (Amin et al., 1999). Therefore, these data argue against a general important role of cpSRP54 in cotranslational protein insertion, although it cannot be ruled out that the loss of cpSRP54 in the *ffc* mutant is compensated by e.g., the upregulation of stromal chaperones as shown for ClpC (Rutschow et al., 2008). The present report extended the analysis of the role of cpSRP54 in cotranslational protein insertion by focusing on the PS II repair cycle during high light conditions. As it seems rather unlikely that the lack of cpSRP can be almost fully compensated by an upregulation of an alternative mechanisms under these stress conditions, our data indicate that cpSRP54 has no critical role in PS II maintenance. However, as there are several lines of evidence indicating a role of cpSRP54 in D1 biogenesis it might be possible that cpSRP54 has its major role in the *de novo* synthesis of photosynthetic protein complexes during leaf development.

Our data reveal a drastic disturbance of the PS II repair cycle due to the lack of the SRP-receptor cpFtsY. While the common model of bacterial SRP-mediated protein insertion proposes that ribosomes and transcripts are cotargeted to the membrane, where FtsY mediates the docking of the SRP-RNC complex to the SecYEG translocase (Luirink et al., 2005; Akopian et al., 2013), an alternative model is discussed in which ribosomes and transcripts are targeted separately to the membrane (Bibi, 2012). Here, FtsY serves as an anchor for ribosomes at the plasma membrane. Inner membrane protein coding mRNAs are delivered to the ribosome in a translation independent manner. Afterwards, the translation process is initiated. In a following step, SRP binds to the nascent chain and facilitates the insertion of the membrane protein via the Sec-translocase (Bibi, 2012). This model is in line with the finding that FtsY mutants exhibit a reduced number of membrane-associated ribosomes, while this was not observed in Ffh-depleted cells (Herskovits and Bibi, 2000). Interestingly, our data also indicate a crucial role of cpFtsY for the association of plastid polysomes with the thylakoid membrane while cpSRP54 seems to be dispensable for this process.

The minor impact of the lack of cpSRP54 might be explained with the hypothesis of a mRNA-based protein targeting. This mechanism is discussed to be potentially involved in targeting of proteins to the subcellular compartments (Nevo-Dinur et al., 2011; Weis et al., 2013). Likewise, the localization of the *psbA* mRNA at the thylakoid membrane might be important for protein delivery. Several *psbA* mRNA binding proteins have been described in *A. thaliana* and *Chlamydomonas reinhardtii* that are essential for D1 translation and possibly attach the *psbA* mRNA to the thylakoid membrane (Mulo et al., 2012). Another strong evidence for a mRNA-based targeting of D1 was found in *C. reinhardtii*. There, components of the translation machinery and the *psbA* mRNA accumulate in a specific region, called translation zone, independently of translation processes (Uniacke and Zerges, 2007, 2009).

In conclusion, our data indicate an almost cpSRP54-independent targeting and insertion of D1 during the repair cycle of PS II, while cpFtsY plays an important role in this process. It is tempting to speculate that ribosomes might be associated with cpFtsY at the thylakoid membrane and that the *psbA* mRNA is delivered to the ribosomes in a translation-independent mechanism. However, it will be a major task for the future to elucidate the molecular details of cotranslational D1 insertion.

## Author contributions

DS and BW designed the research. BW and TP performed research. All authors contributed to data analyses and manuscript preparation.

### Conflict of interest statement

The authors declare that the research was conducted in the absence of any commercial or financial relationships that could be construed as a potential conflict of interest.

## Abbreviations

PS II: photosystem II
RNC: ribosome nascent chain complex
SRP: signal recognition particle.

## References

[B1] Akopian D. Shen K. Zhang X. Shan S. O. (2013). Signal recognition particle: an essential protein-targeting machine. Annu. Rev. Biochem. 82, 693–721. 10.1146/annurev-biochem-072711-164732 23414305 PMC3805129

[B2] Albiniak A. M. Baglieri J. Robinson C. (2012). Targeting of lumenal proteins across the thylakoid membrane. J. Exp. Bot. 63, 1689–1698. 10.1093/jxb/err444 22275386

[B3] Amin P. Sy D. A. Pilgrim M. L. Parry D. H. Nussaume L. Hoffman N. E. (1999). Arabidopsis mutants lacking the 43- and 54-kilodalton subunits of the chloroplast signal recognition particle have distinct phenotypes. Plant Physiol. 121, 61–70. 10.1104/pp.121.1.61 10482661 PMC59390

[B4] Asakura Y. Hirohashi T. Kikuchi S. Belcher S. Osborne E. Yano S.. (2004). Maize mutants lacking chloroplast FtsY exhibit pleiotropic defects in the biogenesis of thylakoid membranes. Plant Cell 16, 201–214. 10.1105/tpc.014787 14688289 PMC301405

[B5] Asakura Y. Kikuchi S. Nakai M. (2008). Non-identical contributions of two membrane-bound cpSRP components, cpFtsY and Alb3, to thylakoid biogenesis. Plant J. 56, 1007–1017. 10.1111/j.1365-313X.2008.03659.x 18764927

[B6] Barkan A. (1993). Nuclear mutants of maize with defects in chloroplast polysome assembly have altered chloroplast RNA metabolism. Plant Cell 5, 389–402. 10.1105/tpc.5.4.389 12271069 PMC160279

[B7] Bibi E. (2012). Is there a twist in the *Escherichia coli* signal recognition particle pathway? Trends Biochem. Sci. 37, 1–6. 10.1016/j.tibs.2011.09.004 22088262

[B8] Bornemann T. Jockel J. Rodnina M. V. Wintermeyer W. (2008). Signal sequence-independent membrane targeting of ribosomes containing short nascent peptides within the exit tunnel. Nat. Struct. Mol. Biol. 15, 494–499. 10.1038/nsmb.1402 18391966

[B9] Celedon J. M. Cline K. (2013). Intra-plastid protein trafficking: how plant cells adapted prokaryotic mechanisms to the eukaryotic condition. Biochim. Biophys. Acta 1833, 341–351 10.1016/j.bbamcr.2012.06.028 22750312 PMC3481018

[B10] Denks K. Vogt A. Sachelaru I. Petriman N. A. Kudva R. Koch H. G. (2014). The Sec translocon mediated protein transport in prokaryotes and eukaryotes. Mol. Membr. Biol. 31, 58–84. 10.3109/09687688.2014.907455 24762201

[B11] Franklin A. E. Hoffman N. E. (1993). Characterization of a chloroplast homologue of the 54-kDa subunit of the signal recognition particle. J. Biol. Chem. 268, 22175–22180. 8408079

[B12] Gu S. Q. Peske F. Wieden H. J. Rodnina M. V. Wintermeyer W. (2003). The signal recognition particle binds to protein L23 at the peptide exit of the *Escherichia coli* ribosome. RNA 9, 566–573. 10.1261/rna.2196403 12702815 PMC1370422

[B13] Herskovits A. A. Bibi E. (2000). Association of *Escherichia coli* ribosomes with the inner membrane requires the signal recognition particle receptor but is independent of the signal recognition particle. Proc. Natl. Acad. Sci. U.S.A. 97, 4621–4626. 10.1073/pnas.080077197 10781067 PMC18282

[B14] Kato Y. Sakamoto W. (2009). Protein quality control in chloroplasts: a current model of D1 protein degradation in the photosystem II repair cycle. J. Biochem. 146, 463–469. 10.1093/jb/mvp073 19451147

[B15] Klimyuk V. I. Persello-Cartieaux F. Havaux M. Contard-David P. Schuenemann D. Meiherhoff K.. (1999). A chromodomain protein encoded by the arabidopsis CAO gene is a plant- specific component of the chloroplast signal recognition particle pathway that is involved in LHCP targeting. Plant Cell 11, 87–99. 10.1105/tpc.11.1.87 9878634 PMC144089

[B16] Lennartz K. Plucken H. Seidler A. Westhoff P. Bechtold N. Meierhoff K. (2001). HCF164 encodes a thioredoxin-like protein involved in the biogenesis of the cytochrome b(6)f complex in Arabidopsis. Plant Cell 13, 2539–2551. 10.2307/3871593 11701887 PMC139470

[B17] Loschelder H. Schweer J. Link B. Link G. (2006). Dual temporal role of plastid sigma factor 6 in Arabidopsis development. Plant Physiol. 142, 642–650. 10.1104/pp.106.085878 16905663 PMC1586057

[B18] Lu B. Hanson M. R. (1996). Fully edited and partially edited nad9 transcripts differ in size and both are associated with polysomes in potato mitochondria. Nucleic Acids Res. 24, 1369–1374. 10.1093/nar/24.7.1369 8614643 PMC145777

[B19] Luirink J. von Heijne G. Houben E. de Gier J. W. (2005). Biogenesis of inner membrane proteins in *Escherichia coli*. Annu. Rev. Microbiol. 59, 329–355. 10.1146/annurev.micro.59.030804.121246 16153172

[B20] Mulo P. Sakurai I. Aro E. M. (2012). Strategies for psbA gene expression in cyanobacteria, green algae and higher plants: from transcription to PSII repair. Biochim. Biophys. Acta 1817, 247–257. 10.1016/j.bbabio.2011.04.011 21565160

[B21] Nevo-Dinur K. Nussbaum-Shochat A. Ben-Yehuda S. Amster-Choder O. (2011). Translation-independent localization of mRNA in *E. coli.* Science 331, 1081–1084. 10.1126/science.1195691 21350180

[B22] Nilsson R. Brunner J. Hoffman N. E. van Wijk K. J. (1999). Interactions of ribosome nascent chain complexes of the chloroplast- encoded D1 thylakoid membrane protein with cpSRP54. EMBO J. 18, 733–742. 10.1093/emboj/18.3.733 9927433 PMC1171166

[B23] Nilsson R. van Wijk K. J. (2002). Transient interaction of cpSRP54 with elongating nascent chains of the chloroplast-encoded D1 protein; ‘cpSRP54 caught in the act.’ FEBS Lett. 524, 127–133. 10.1016/S0014-5793(02)03016-8 12135754

[B24] Nixon P. J. Michoux F. Yu J. Boehm M. Komenda J. (2010). Recent advances in understanding the assembly and repair of photosystem II. Ann. Bot. 106, 1–16. 10.1093/aob/mcq059 20338950 PMC2889791

[B25] Paila Y. D. Richardson L. G. Schnell D. J. (2014). New insights into the mechanism of chloroplast protein import and its integration with protein quality control, organelle biogenesis and development. J. Mol. Biol. 427, 1038–1060. 10.1016/j.jmb.2014.08.016 25174336 PMC4339491

[B26] Richter C. V. Bals T. Schunemann D. (2010). Component interactions, regulation and mechanisms of chloroplast signal recognition particle-dependent protein transport. Eur. J. Cell Biol. 89, 965–973. 10.1016/j.ejcb.2010.06.020 20709425

[B27] Röhl T. van Wijk K. J. (2001). *In vitro* reconstitution of insertion and processing of cytochrome f in a homologous chloroplast translation system. J. Biol. Chem. 276, 35465–35472. 10.1074/jbc.M103005200 11459839

[B28] Rutschow H. Ytterberg A. J. Friso G. Nilsson R. van Wijk K. J. (2008). Quantitative proteomics of a chloroplast SRP54 sorting mutant and its genetic interactions with CLPC1 in Arabidopsis. Plant Physiol. 148, 156–175. 10.1104/pp.108.124545 18633119 PMC2528104

[B29] Schagger H. von Jagow G. (1991). Blue native electrophoresis for isolation of membrane protein complexes in enzymatically active form. Anal. Biochem. 199, 223–231. 10.1016/0003-2697(91)90094-A 1812789

[B30] Schleiff E. Becker T. (2011). Common ground for protein translocation: access control for mitochondria and chloroplasts. Nat. Rev. Mol. Cell Biol. 12, 48–59. 10.1038/nrm3027 21139638

[B31] Schult K. Meierhoff K. Paradies S. Toller T. Wolff P. Westhoff P. (2007). The nuclear-encoded factor HCF173 is involved in the initiation of translation of the psbA mRNA in *Arabidopsis thaliana*. Plant Cell 19, 1329–1346. 10.1105/tpc.106.042895 17435084 PMC1913763

[B32] Schünemann D. (2007). Mechanisms of protein import into thylakoids of chloroplasts. Biol. Chem. 388, 907–915. 10.1515/BC.2007.111 17696774

[B33] Schünemann D. Gupta S. Persello-Cartieaux F. Klimyuk V. I. Jones J. D. Nussaume L.. (1998). A novel signal recognition particle targets light-harvesting proteins to the thylakoid membranes. Proc. Natl. Acad. Sci. U.S.A. 95, 10312–10316. 10.1073/pnas.95.17.10312 9707644 PMC21505

[B34] Seluanov A. Bibi E. (1997). FtsY, the prokaryotic signal recognition particle receptor homologue, is essential for biogenesis of membrane proteins. J. Biol. Chem. 272, 2053–2055. 10.1074/jbc.272.4.2053 8999901

[B35] Träger C. Rosenblad M. A. Ziehe D. Garcia-Petit C. Schrader L. Kock K. (2012). Evolution from the prokaryotic to the higher plant chloroplast signal recognition particle: the signal recognition particle RNA is conserved in plastids of a wide range of photosynthetic organisms. Plant Cell 24, 4819–4836 10.1105/tpc.112.102996 23275580 PMC3556960

[B36] Tzvetkova-Chevolleau T. Hutin C. Noel L. D. Goforth R. Carde J. P. Caffarri S. (2007). Canonical signal recognition particle components can be bypassed for posttranslational protein targeting in chloroplasts. Plant Cell 19, 1635–1648 10.1105/tpc.106.048959 17513500 PMC1913721

[B37] Uniacke J. Zerges W. (2007). Photosystem II assembly and repair are differentially localized in Chlamydomonas. Plant Cell 19, 3640–3654. 10.1105/tpc.107.054882 18055604 PMC2174875

[B38] Uniacke J. Zerges W. (2009). Chloroplast protein targeting involves localized translation in Chlamydomonas. Proc. Natl. Acad. Sci. U.S.A. 106, 1439–1444. 10.1073/pnas.0811268106 19164529 PMC2629442

[B39] Weis B. L. Schleiff E. Zerges W. (2013). Protein targeting to subcellular organelles via MRNA localization. Biochim. Biophys. Acta 1833, 260–273. 10.1016/j.bbamcr.2012.04.004 23457718

[B40] Yamamoto T. Shimizu Y. Ueda T. Shiro Y. (2010). Mg2+ dependence of 70 s ribosomal protein flexibility revealed by hydrogen/deuterium exchange and mass spectrometry. J. Biol. Chem. 285, 5646–5652. 10.1074/jbc.M109.081836 20022945 PMC2820792

[B41] Zhang L. X. Paakkarinen V. Suorsa M. Aro E. M. (2001). A SecY homologue is involved in chloroplast-encoded D1 protein biogenesis. J. Biol. Chem. 276, 37809–37814. 10.1074/jbc.M105522200 11473124

